# Common Cancer-Related Factors and the Risk of Developing Kaposi Sarcoma in Individuals without AIDS: Korea National Health Insurance Services Claims Database

**DOI:** 10.3390/jcm13185634

**Published:** 2024-09-23

**Authors:** Ji Eun Shin, Kyungdo Han, Ho Jung An, Hyung Soon Park, Byoung Yong Shim, Hyunho Kim

**Affiliations:** 1 Division of Medical Oncology, Department of Internal Medicine, St. Vincent’s Hospital, College of Medicine, The Catholic University of Korea, 93 Jungbu-daero, Paldal-gu, Suwon 16247, Republic of Korea; jieun.shin.onco@gmail.com (J.E.S.); strhojung@gmail.com (H.J.A.); happymeruk@gmail.com (H.S.P.); shimby@catholic.ac.kr (B.Y.S.); 2 Department of Statistics and Actuarial Science, Soongsil University, 369, Sangdo-ro, Dongjak-gu, Seoul 06978, Republic of Korea; hkd917@naver.com

**Keywords:** Kaposi sarcoma, incidence, risk factor

## Abstract

**Backgrounds:** Kaposi sarcoma (KS) is a unique form of cancer with epidemiological characteristics distinct from those of other solid cancers. While common risk factors including alcohol consumption, smoking, and metabolic disorders have been well studied in various cancers, their relationship with KS remains unclear. **Methods:** This study used a cohort approach with adults without AIDS, utilizing data from the National Health Insurance Service in South Korea. This study examined various conventional cancer-related risk factors related to the incidence of KS, including psoriasis. **Results:** Alcohol consumption, smoking, body mass index, diabetes mellitus, hypertension, hypercholesterolemia, and regular exercise were not significantly associated with the incidence of KS. Additionally, older age and male sex were associated with a higher incidence of KS. KS risk was increased in pathological conditions such as psoriasis and proteinuria, which require immunosuppressive medication. **Conclusions:** Our study suggests that traditional cancer-related risk factors may not play a significant role in the pathogenesis of KS, unlike other cancers. This, in turn, emphasizes the importance of immunosuppression and HHV-8 infection in the development of KS.

## 1. Introduction

Kaposi sarcoma (KS) is a rare neoplasm primarily originating from the endothelial cell lineage of the blood or lymphatic vessels [1]. It is most commonly observed on the skin or mucosal surfaces from the mouth to the anus [2]. Although generally considered an indolent tumor, it can occasionally manifest aggressively, especially when visceral organ involvement is present, resulting in a wide range of clinical presentations [2].

The etiology of KS is closely linked to a compromised systemic immune system and infection by Kaposi’s sarcoma-associated herpesvirus (HHV-8), which was designated as a class I carcinogen by the International Agency for Research on Cancer/World Health Organization in 2009 [1,2]. Consequently, human immunodeficiency virus (HIV)-associated KS is a major epidemiological form of this disease [3]. In the United States, KS incidence dramatically increased in the 1980s alongside the spread of AIDS but saw a sharp decline in the 1990s following the advent of antiviral therapies for HIV infection [4].

There are several types of non-HIV-associated KS: “Classic”, which is associated with people from the Mediterranean region; “Endemic”, which is prevalent among people in sub-Saharan Africa; and “Iatrogenic”, which occurs in individuals who are immunosuppressed, such as solid organ transplant recipients. Thus, Kaposi sarcoma (KS) is a unique form of cancer with epidemiological characteristics distinct from those of other solid cancers [5].

In the case of non-HIV-associated KS, KS is influenced by several risk factors, particularly those related to immunosuppression. One of the most significant risk factors is the use of immunosuppressive therapy, especially in organ transplant recipients, where prolonged immunosuppressive treatment increases the risk of KS development. Additionally, older age and male gender are well-established risk factors for the classic form of KS, particularly among individuals from Mediterranean regions, where the disease typically progresses slowly. Environmental factors, such as exposure to volcanic soil, have also been hypothesized as potential contributors to KS risk, particularly in endemic regions [5,6].

However, while common risk factors such as alcohol consumption, smoking, and metabolic disorders have been well-studied in various cancers, their relationship with KS has been less explored. This may be due to the rarity of the disease and the indolent nature, making it challenging to recruit a sufficient number of research subjects from a single institution for risk factor analysis [7,8].

Therefore, the aim of our study was to analyze the risk factors associated with the development of KS using national health screening data from a large cohort of individuals not affected by AIDS.

## 2. Materials and Methods

### 2.1. Database and Study Population

The National Health Insurance Service (NHIS), administered by the Korean government, covers approximately 97% of the total population through a mandatory health insurance system [9]. The NHIS conducts biannual routine health checkups targeting all insured individuals aged 40 years and above and employed individuals aged 20 years and above [9]. The examination data include factors related to metabolic syndrome, such as hypertension (HTN), diabetes mellitus (DM), dyslipidemia, and obesity, and lifestyle-related factors, including smoking, alcohol consumption, and exercise. In addition, the NHIS provides a comprehensive set of baseline demographic and clinical variables of enrollees, including age, sex, income rank, utilization of inpatient and outpatient services, and diagnostic codes.

Such foundational data, along with health screening metrics, are ascertained at the time of each subject’s enrollment in the health screening program. Our study was conducted on individuals who participated in the NHIS National Health Screening Program between 1 January 2009, and 31 December 2009. The endpoint for follow-up observations was 31 December 2018.

### 2.2. Operational Definition of Kaposi Sarcoma and Other Diseases

KS was defined as having at least two claims under the International Statistical Classification of Diseases, 10th Revision, Clinical Modification (ICD-10-CM) code C46. To accurately identify patients diagnosed with KS, we utilized a special reimbursement code program (V code) implemented by the NHIS to reduce the financial burden on cancer patients [9]. As medical certification by a physician is required for this program, we identified cases of KS diagnosed during the follow-up period using both the ICD-10-CM code C46 and a cancer-specific reimbursement code (V code: V193). HIV infection or AIDS is defined as cases that have been hospitalized at least once or have received outpatient treatment more than twice under the ICD-10-CM codes B20 or B24. Atopic dermatitis, dry skin, urticaria, and psoriasis are, respectively, defined as cases that have been hospitalized at least once or have received outpatient treatment more than twice under the ICD-10-CM codes L20, L29.8 and L85.3, L50, and L40.

### 2.3. Definition of Other Variables

Age was defined as the baseline age at the time of enrollment. Body mass index (BMI) was determined by dividing weight in kilograms by the square of height in meters. DM was characterized by a fasting glucose level equal to or greater than 126 mg/dL or a minimum of one annual claim for a prescription of antidiabetic medication, identified through ICD-10-CM codes E11–E14. Dyslipidemia was determined based on total cholesterol levels equal to or exceeding 240 mg/dL or the presence of prescriptions for lipid-lowering drugs, identified under ICD-10-CM code E78. HTN was defined as systolic blood pressure equal to or greater than 140 mmHg, diastolic blood pressure equal to or greater than 90 mmHg, or the presence of an antihypertensive drug prescription under ICD-10-CM codes I10-I13 and I15. Waist circumference was measured at the midpoint between the lower margin of the last palpable rib and the top of the iliac crest. Biomarkers, including fasting glucose, total cholesterol, triglycerides, LDL-C, and HDL-C, were obtained from serum or plasma samples collected at the initiation of the health screening program. Alcohol consumption was stratified into non-drinkers, mild drinkers (0–30 g/day), and heavy drinkers (>30 g/day). Physical activity was categorized based on frequency and intensity: either no exercise (<3 days per week of vigorous intensity and <5 days per week of moderate intensity) or exercise (≥3 days per week of vigorous intensity or ≥5 days per week of moderate intensity). Income was divided into quartiles, where Q1 represented the lowest income bracket and Q4 represented the highest.

### 2.4. Statistical Analysis

Baseline characteristics are presented as the mean and standard deviation, or a number (%). Categorical variables were compared using the chi-square test. Continuous variables were compared using Student’s t-test. Incidence rates for KS were computed by dividing the number of events by the total follow-up durations, and the results were expressed as the rate per 1000 person-years. Participants were observed throughout the follow-up period until the occurrence of the first diagnosis of KS, death, or 31 December 2018, whichever took place first. Multivariable Cox proportional hazards regression models were used to calculate hazard ratios (HRs) and the corresponding 95% confidence intervals (CIs). Model 1 was constructed without any adjustments. Model 2 was adjusted for age and sex. Model 3 was adjusted for age, sex, BMI, income, smoking, drinking, and exercise. Model 4 was adjusted for age, sex, BMI, income, smoking, drinking, exercise, DM, HTN, and dyslipidemia. Statistical significance was set at a two-sided *p* value < 0.05. All the statistical analyses were performed using SAS, version 9.3 (SAS Institute, Cary, NC, USA).

## 3. Results

### 3.1. Study Participants

**Figure 1:**
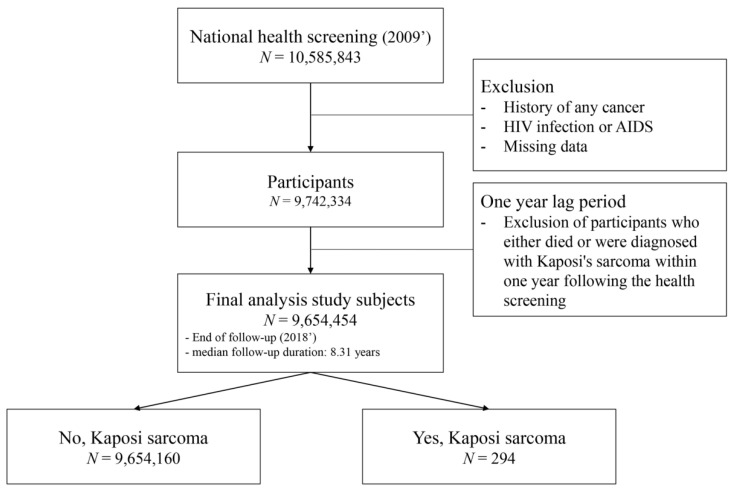
Study design and participants.

A total of 10,585,843 adults aged 20 and above who underwent health screening in 2009 were initially enrolled in this study. Of these, 162,512 individuals who were diagnosed with or treated for cancer and 913 patients with HIV or AIDS were excluded from the analysis. Subsequently, individuals with missing data (N = 680,084) were removed from the study cohort. To enhance the explanatory power regarding causality, participants who either died within one year following health screening or were diagnosed with KS within the same one-year period (N = 87,880) were also excluded. Ultimately, the study population consisted of 9,654,454 individuals (Figure 1).

### 3.2. Baseline Characteristics of the Study Participants

During a median follow-up duration of 8.3 years, 294 participants were diagnosed with KS. The baseline characteristics of the study participants, both those diagnosed with KS and those who were not, are described in Table 1. Factors such as older age, smoking, alcohol consumption, DM, HTN, dyslipidemia, chronic kidney disease (CKD), and dermatological conditions, including psoriasis, atopic dermatitis, dry skin, and urticaria, were more prevalent among participants who developed KS. The mean age, BMI, waist circumference, fasting glucose level, and systolic blood pressure were higher in those who developed KS than in those who did not. There were no significant differences in the mean values of total cholesterol, HDL-C, LDL-C, or triglycerides between the two groups.

### 3.3. Analysis of Kaposi Sarcoma Risk in Non-AIDS Adults

To identify risk factors associated with the incidence of KS in healthy adults aged 20 and above without AIDS or HIV infection, we conducted multiple regression analyses, with HRs and 95% CIs delineated in Table 2. In the univariate analysis, the risk ratios for KS varied depending on age, sex, income, smoking status, alcohol consumption, DM, HTN, dyslipidemia, CKD, proteinuria, and psoriasis. However, when these variables were incorporated into the multivariate analysis, only older age, male sex, and psoriasis remained statistically significant factors contributing to an elevated risk of KS. Specifically, in subjects with psoriasis, the HR was notably high at 3.572 (95% CI 1.768–7.219).

Gender-specific subgroup analysis showed that in males, older age, obesity, the presence of urinary protein, and psoriasis significantly increased the risk of KS, with HRs of 1.075, 2.307, 2.141, and 3.481, respectively. In females, older age increased the risk. No other factors were statistically significant.

In the age-specific subgroup analysis of those aged >65 years, the risk of developing KS was higher in males and those with psoriasis, with psoriasis showing a remarkably high HR of 4.734. For those under 65 years of age, no factor yielded statistically significant HRs.

Given that psoriasis consistently emerged as a significant risk factor for the onset of KS in both the overall patient cohort and in age- and sex-specific subgroup analyses, we extended our investigation to include common skin disorders such as atopic dermatitis, dry skin, and urticaria (Table 3). In the non-adjusted analysis (Model 1), the HRs for the incidence of KS in adults with dry skin, urticaria, and psoriasis, excluding atopic dermatitis, were significantly elevated at 2.835 (95% CI 1.263–6.363), 1.913 (95% CI 1.202–3.045), and 4.346 (95% CI 2.153–8.774), respectively. Interestingly, upon additional adjustment for variables such as age, sex, BMI, income, smoking status, alcohol consumption, exercise, DM, HTN, and dyslipidemia (Model 4), only psoriasis remained a statistically significant risk factor, with an adjusted HR of 3.569 (95% CI 1.766–7.213). In contrast, the adjusted HRs (95% CIs) for atopic dermatitis, dry skin, and urticaria were 1.248 (0.400–3.896), 1.846 (0.821–4.152), and 1.460 (0.915–2.329), respectively. However, these were not statistically significant.

## 4. Discussion

Kaposi sarcoma (KS) is distinctly caused by HHV-8 infection, with a high prevalence observed in specific regions such as sub-Saharan Africa and the Mediterranean basin [5]. While sexual contact, particularly among men who have sex with men, is a known transmission route of HHV-8, it does not account for all infection pathways [5]. In regions like East Asia, where HHV-8 prevalence is low, KS typically occurs in individuals with immunosuppressive conditions such as those undergoing organ transplantation or prolonged steroid use [10]. However, the association between KS and common cancer risk factors such as smoking, alcohol consumption, obesity, and metabolic diseases remains underexplored. This gap in information, especially in regions like East Asia where KS is rare, is primarily due to the rarity of KS [7]. Our study, involving approximately ten million Korean adults without AIDS, addresses this issue on a relatively large scale. We found no significant association between KS incidence and commonly known cancer-related factors such as smoking, alcohol consumption, obesity, physical activity, and metabolic diseases. Consistent with existing knowledge, we observed that the frequency of HIV-unrelated KS was higher among males and those of advanced age [2].

Both alcohol consumption and smoking are well-known carcinogens associated with the risk of developing various solid cancers [11,12]. However, in our study, neither alcohol consumption nor smoking was significantly associated with the risk of KS. This was consistent even in subgroup analyses stratified by age and sex. Although not directly related to the risk of KS, a previous epidemiological study suggested that alcohol consumption may elevate the risk of HHV-8 infection [13]. However, another study reported no increased risk associated with alcohol consumption or smoking [14]. Direct associations between alcohol-drinking behavior or smoking and the risk of developing KS have been sparsely studied.

Previous studies have reported an association between metabolic syndrome and the risk of relatively common cancers such as liver, colorectal, bladder, endometrial, and breast cancers in women [15,16]. Diagnostic criteria for metabolic syndrome include factors related to DM, HTN, dyslipidemia, and obesity. Specifically, conditions such as DM are known to have a strong correlation with immunity and influence various systemic infections [17]. Case–control studies have reported an association between HHV-8 infection and the risk of developing KS in individuals with DM [13]. However, in our study, while DM appeared to be relevant in the univariate analysis, its association with the risk of developing KS was nullified in the multivariate analysis. Similarly, hypertension (HTN) and dyslipidemia were also significant only in the univariate analysis. This is likely related to confounding effects of age. The National Health and Nutrition Examination Survey (NHANES) data from 2011 to 2016 reported that the prevalence of metabolic syndrome increases with age [18].

Diseases related to metabolic syndrome, such as DM, HTN, and obesity, can be modified through lifestyle adjustments, such as regular exercise, a healthy diet, and appropriate weight management. Previous reports have demonstrated that maintaining a healthy weight through regular physical activity and dietary modifications can effectively reduce the risk of cancer [16]. Although these reports did not specifically include studies on KS, they encompassed a wide range of cancer types, including head and neck, stomach, colorectal, liver, pancreatic, lung, breast, endometrial, and bladder cancer [16]. In contrast, our study did not show any protective effect of regular exercise. Given the presence of various confounding factors, caution is warranted in interpretation. However, based on our findings, it can be inferred that common cancer-related factors such as alcohol consumption, smoking, diabetes mellitus, hypertension, hypercholesterolemia, obesity, and lifestyle are unlikely to significantly increase the risk of HHV-8 infection, the primary cause of KS.

Within the available dataset, we randomly selected several diseases of interest to analyze their association with KS incidence. Notably, psoriasis showed a significantly high hazard ratio (HR). The curiosity about KS risk in diseases related to T cell immunity, such as psoriasis, has been suggested in the past [19,20]. In particular, pro-inflammatory cytokines, including interleukin-6, play a significant role in the pathogenesis of both psoriasis and KS [21,22]. However, an Italian study argued that there was no association between psoriasis and development of KS, reporting that the prevalence of psoriasis was approximately 2% in both the general and KS populations [19]. Additionally, they noted a long latency of 18.8 years between the onset of psoriasis and the occurrence of KS [19].

Treatment for psoriasis often includes immunosuppressive agents such as corticosteroids, calcineurin inhibitors, methotrexate, and cyclosporine [23]. Therefore, it is plausible to suspect that the higher incidence of KS in individuals with psoriasis observed in our study might be due to the use of immunosuppressive medications rather than the psoriasis itself. Our results that KS risk did not increase in other skin disorders with less systemic immunosuppressive treatment, such as dry skin and urticaria, supports this hypothesis. Unfortunately, our dataset did not include information on immunosuppressive treatment, preventing us from analyzing this factor further.

In our analysis, individuals with proteinuria had a higher incidence of KS compared to those without proteinuria. Since nephrotic syndrome, a major cause of proteinuria, is treated with immunosuppressive agents [24], it is likely that the observed association between proteinuria and KS risk is due to the use of these medications, similar to psoriasis.

Similar to previous reports, we also found a higher incidence of HIV-unrelated KS among males. In this difference according to gender, metabolic and physiological factors could play a significant role. Studies have shown that male predominance in non-HIV-associated KS cases, particularly in elderly men from Mediterranean regions, may be linked to gender-specific factors such as hormonal influences, immune response variability, and different exposure to environmental factors [25]. Males typically exhibit higher levels of systemic inflammation and angiogenic factors, which are critical in KS development. Moreover, iatrogenic immunosuppression, often required for conditions like organ transplants, appears to disproportionately affect males [26]. The progression of KS in men might also be influenced by metabolic conditions such as hypertension and diabetes, both of which are more prevalent in older males and may exacerbate the angiogenic pathways involved in KS. Therefore, understanding these physiological and metabolic factors is crucial for elucidating the gender disparity in non-HIV-associated KS.

Our study has several limitations: First, it is an uncontrolled, simple observational study, which limits its explanatory power regarding causality. Additionally, owing to the limited data provided by the NHIS, our study did not encompass all potential confounding variables. Importantly, we were unable to obtain prescription data related to immunosuppressive drugs, such as steroids and cyclosporine, preventing us from analyzing iatrogenic KS. Second, the small number of individuals who developed KS compared to the total study population limits the reliability and generalizability of our findings. In particular, only eight individuals with psoriasis developed KS. Third, our findings are based solely on a Korean population, necessitating the need for multicenter multinational studies to substantiate our results.

In conclusion, our study was unable to demonstrate a relationship between commonly acknowledged factors such as alcohol consumption, smoking, BMI, and DM and the risk of developing KS. This, in turn, emphasizes the importance of immunosuppression and HHV-8 infection in the development of KS. It also suggests that common cancer-related factors have a minimal impact on increasing the risk of HHV-8 infection.

## Figures and Tables

**Table 1:** Baseline characteristics of the study population.

	Kaposi Sarcoma (−) *n* = 9,654,160	Kaposi Sarcoma (+) *n* = 294	*p* Value
Age groups			<0.0001
<40	3,041,798 (31.51)	44 (14.97)	
40–64	5,391,007 (55.84)	143 (48.64)	
≥65	1,221,355 (12.65)	107 (36.39)	
Income, lowest Q1	1,885,476 (19.53)	51 (17.35)	0.345
BMI level			0.1006
<18.5 kg/m^2^	356,696 (3.69)	6 (2.04)	
<23 kg/m^2^	3,764,502 (38.99)	112 (38.1)	
<25 kg/m^2^	2,376,910 (24.62)	62 (21.09)	
<30 kg/m^2^	2,812,001 (29.13)	99 (33.67)	
≥30 kg/m^2^	344,051 (3.56)	15 (5.1)	
Smoking			0.0005
Non	5,730,881 (59.36)	172 (58.5)	
Ex	1,376,477 (14.26)	63 (21.43)	
Current	2,546,802 (26.38)	59 (20.07)	
Drinking			0.012
Non	4,944,380 (51.22)	174 (59.18)	
Mild	3,933,363 (40.74)	95 (32.31)	
Heavy	776,417 (8.04)	25 (8.5)	
Regular exercise	1,725,329 (17.87)	63 (21.43)	0.1114
Diabetes mellitus	835,624 (8.66)	50 (17.01)	<0.0001
Hypertension	2,479,886 (25.69)	127 (43.2)	<0.0001
Dyslipidemia	1,744,330 (18.07)	74 (25.17)	0.0016
CKD	660,037 (6.84)	30 (10.2)	0.0222
Urine protein, positive	242,871 (2.52)	15 (5.1)	0.0046
Migraine	265,927 (2.75)	12 (4.08)	0.1644
Depression	307,420 (3.18)	10 (3.4)	0.8322
Psoriasis	62,302 (0.65)	8 (2.72)	<0.0001
Atopic dermatitis	68,554 (0.71)	3 (1.02)	0.5263
Dry skin	72,186 (0.75)	6 (2.04)	0.0101
Urticaria	340,063 (3.52)	19 (6.46)	0.0062
Age, years	46.99 ± 13.96	57.38 ± 14.71	<0.0001
Height, cm	163.9 ± 9.24	162.67 ± 8.52	0.0231
Weight, kg	63.94 ± 11.64	64.27 ± 12	0.6296
BMI, kg/m^2^	23.71 ± 3.23	24.18 ± 3.34	0.0129
Waist circumference, cm	80.24 ± 9.11	82.67 ± 9.78	<0.0001
Fasting glucose, mg/dL	97.26 ± 23.85	100.97 ± 24.64	0.0077
Systolic BP, mmHg	122.45 ± 15.05	125.95 ± 17.15	<0.0001
Diastolic BP, mmHg	76.33 ± 10.07	76.8 ± 10.75	0.4286
Total cholesterol, mg/dL	195.15 ± 36.9	194.82 ± 41.84	0.8784
HDL-C, mg/dL	56.1 ± 27.93	54.58 ± 25.83	0.3527
LDL-C, mg/dL	113.53 ± 38.67	113.06 ± 36.56	0.8358
Triglyceride, mg/dL	112.82 (112.77–112.86)	117.82 (110.37–125.77)	0.2004

Data are shown as mean ± standard deviation, median (25–75th percentile), or number (%). Abbreviations: BMI—body mass index; BP—blood pressure; CKD—chronic kidney disease; HDL—high-density lipoprotein; LDL—low-density lipoprotein.

**Table 2:** Subgroup analysis of the association between Kaposi sarcoma risk and various factors.

	Total								Male		Female		Age < 65 Years		Age ≥ 65 Years	
	N	Event	Duration, PY	IR, per 1000 PY	Univariate HR	*p*-Value	Multivariate HR	*p*-Value	Multivariate HR	*p*-Value	Multivariate HR	*p*-Value	Multivariate HR	*p*-Value	Multivariate HR	*p*-Value
Age, years					1.056 (1.048, 1.065)	<0.0001	1.055 (1.045, 1.065)	<0.0001	1.075 (1.061, 1.088)	<0.0001	1.025 (1.007, 1.042)	0.0052	1.043 (1.027, 1.060)	<0.0001	1.065 (1.026, 1.105)	0.001
Sex						0.0085		0.0002						0.7648		<0.0001
Male	5,290,997	183	43,343,918.43	0.004222046	1.372 (1.084, 1.737)		1.788 (1.312, 2.436)						0.938 (0.619, 1.424)		4.811 (2.894, 7.998)	
Female	4,363,457	111	36,132,818.1	0.003072	1 (Ref.)		1 (Ref.)						1 (Ref.)		1 (Ref.)	
Income						0.0397		0.365		0.6723		0.1677		0.2042		0.8549
MA, Q1	1,885,527	51	15,469,974.89	0.003296709	1 (Ref.)		1 (Ref.)		1 (Ref.)		1 (Ref.)		1 (Ref.)		1 (Ref.)	
Q2	2,225,329	51	18,333,674.38	0.002781766	0.843 (0.572, 1.243)		1.018 (0.690, 1.502)		1.041 (0.634, 1.709)		1.043 (0.556, 1.955)		0.952 (0.593, 1.530)		1.256 (0.634, 2.488)	
Q3	2,649,642	88	21,854,552.33	0.004026621	1.222 (0.865, 1.725)		1.305 (0.923, 1.844)		1.243 (0.799, 1.934)		1.664 (0.954, 2.901)		1.409 (0.926, 2.146)		1.243 (0.670, 2.304)	
Q4	2,893,956	104	23,818,534.93	0.004366347	1.327 (0.949, 1.855)		1.105 (0.789, 1.547)		1.015 (0.661, 1.560)		1.545 (0.894, 2.671)		1.099 (0.713, 1.694)		1.275 (0.730, 2.226)	
BMI level						0.1133		0.1268		0.0334		0.5473		0.0991		0.2201
<18.5 kg/m^2^	356,702	6	2,884,634.98	0.002079986	0.575 (0.253, 1.308)		0.716 (0.315, 1.632)		0.922 (0.334, 2.549)		0.389 (0.094, 1.603)		0.579 (0.181, 1.845)		0.790 (0.243, 2.567)	
<23 kg/m^2^	3,764,614	112	30,957,135.16	0.003617906	1 (Ref.)		1 (Ref.)		1 (Ref.)		1 (Ref.)		1 (Ref.)		1 (Ref.)	
<25 kg/m^2^	2,376,972	62	19,604,654.35	0.003162514	0.875 (0.641, 1.193)		0.716 (0.523, 0.979)		0.791 (0.526, 1.190)		0.749 (0.454, 1.234)		0.784 (0.531, 1.159)		0.708 (0.415, 1.207)	
<30 kg/m^2^	2,812,100	99	23,195,951.36	0.004267986	1.180 (0.901, 1.547)		0.927 (0.700, 1.228)		1.177 (0.821, 1.687)		0.792 (0.490, 1.281)		0.871 (0.603, 1.258)		1.259 (0.802, 1.975)	
≥30 kg/m^2^	344,066	15	2,834,360.69	0.005292199	1.463 (0.853, 2.507)		1.346 (0.774, 2.340)		2.307 (1.186, 4.489)		0.757 (0.268, 2.141)		1.743 (0.959, 3.168)		0.388 (0.053, 2.856)	
Smoking						0.0006		0.1947		0.5242		0.5076		0.582		0.2656
Non	5,731,053	172	47,322,573.57	0.003634629	1 (Ref.)		1 (Ref.)		1 (Ref.)		1 (Ref.)		1 (Ref.)		1 (Ref.)	
Ex	1,376,540	63	11,295,763.81	0.005577312	1.533 (1.149, 2.046)		1.156 (0.821, 1.627)		1.106 (0.777, 1.574)		1.890 (0.594, 6.018)		1.280 (0.791, 2.070)		1.127 (0.696, 1.825)	
Current	2,546,861	59	20,858,399.16	0.002828597	0.776 (0.577, 1.043)		0.828 (0.581, 1.180)		0.889 (0.611, 1.294)		1.297 (0.468, 3.596)		1.075 (0.684, 1.691)		0.653 (0.347, 1.231)	
Drinking						0.0102		0.4518		0.4326		0.8959		0.9409		0.2125
Non	4,944,554	174	40,642,358.53	0.004281248	1 (Ref.)		1 (Ref.)		1 (Ref.)		1 (Ref.)		1 (Ref.)		1 (Ref.)	
Mild	3,933,458	95	32,473,135.95	0.002925495	0.680 (0.530, 0.874)		0.838 (0.632, 1.113)		0.813 (0.587, 1.125)		0.884 (0.529, 1.479)		0.964 (0.682, 1.363)		0.645 (0.394, 1.055)	
Heavy	776,442	25	6,361,242.06	0.00393005	0.916 (0.602, 1.393)		0.971 (0.617, 1.529)		0.967 (0.605, 1.545)		0.947 (0.127, 7.047)		1.053 (0.597, 1.858)		0.936 (0.440, 1.990)	
Regular exercise						0.117		0.6887		0.8651		0.8655		0.9806		0.6953
No	7,929,062	231	65,232,946.09	0.003541155	1 (Ref.)		1 (Ref.)		1 (Ref.)		1 (Ref.)		1 (Ref.)		1 (Ref.)	
Yes	1,725,392	63	14,243,790.45	0.00442298	1.250 (0.946, 1.651)		1.059 (0.800, 1.403)		1.030 (0.732, 1.449)		1.044 (0.636, 1.712)		1.005 (0.699, 1.444)		1.094 (0.698, 1.714)	
DM level						<0.0001		0.2433		0.1655		0.6138		0.2643		0.4889
Normal	6,620,422	178	54,728,620.67	0.003252412	1 (Ref.)		1 (Ref.)		1 (Ref.)		1 (Ref.)		1 (Ref.)		1 (Ref.)	
IFG	2,198,358	66	18,058,208.73	0.003654848	1.126 (0.849, 1.493)		0.856 (0.642, 1.142)		0.730 (0.507, 1.052)		1.174 (0.737, 1.872)		0.947 (0.660, 1.359)		0.757 (0.470, 1.220)	
DM	835,674	50	6,689,907.13	0.007473945	2.317 (1.694, 3.171)		1.177 (0.843, 1.645)		1.071 (0.719, 1.593)		1.324 (0.703, 2.492)		1.401 (0.891, 2.205)		0.999 (0.607, 1.644)	
HTN level						<0.0001		0.6322		0.7347		0.7389		0.0702		0.3361
Normal	3,336,731	71	27,679,526.64	0.002565073	1 (Ref.)		1 (Ref.)		1 (Ref.)		1 (Ref.)		1 (Ref.)		1 (Ref.)	
Pre-HTN	3,837,710	96	31,696,612.96	0.003028715	1.182 (0.869, 1.606)		0.943 (0.690, 1.290)		1.114 (0.723, 1.717)		0.954 (0.590, 1.542)		0.879 (0.607, 1.273)		1.305 (0.688, 2.474)	
HTN	2,480,013	127	20,100,596.93	0.00631822	2.483 (1.857, 3.320)		1.085 (0.780, 1.508)		1.194 (0.766, 1.862)		1.162 (0.680, 1.986)		1.355 (0.907, 2.025)		0.947 (0.509, 1.763)	
TC Level						0.0058		0.6432		0.7875		0.5395		0.0909		0.1167
<200	5,182,358	144	42,667,060.02	0.003374969	1 (Ref.)		1 (Ref.)		1 (Ref.)		1 (Ref.)		1 (Ref.)		1 (Ref.)	
<240	2,727,692	76	22,501,596.08	0.003377538	1.001 (0.758, 1.322)		0.876 (0.662, 1.160)		1.021 (0.718, 1.451)		0.768 (0.480, 1.227)		0.668 (0.464, 0.960)		1.583 (1.000, 2.505)	
≥240 or Medication	1,744,404	74	14,308,080.43	0.005171903	1.540 (1.163, 2.038)		0.976 (0.726, 1.311)		1.137 (0.785, 1.647)		0.881 (0.537, 1.444)		0.844 (0.575, 1.240)		1.439 (0.889, 2.330)	
CKD						0.0152		0.9138		0.6549		0.5144		0.9748		0.8933
No	8,994,387	264	74,155,976.45	0.003560064	1 (Ref.)		1 (Ref.)		1 (Ref.)		1 (Ref.)		1 (Ref.)		1 (Ref.)	
Yes	660,067	30	5,320,760.08	0.005638292	1.596 (1.094, 2.329)		0.979 (0.664, 1.442)		0.890 (0.534, 1.484)		1.219 (0.672, 2.213)		0.991 (0.549, 1.787)		1.036 (0.617, 1.741)	
Urine protein						0.0038		0.0859		0.0076		0.2477		0.1399		0.4524
Neg, trace	9,411,568	279	77,530,636.02	0.003598577	1 (Ref.)		1 (Ref.)		1 (Ref.)		1 (Ref.)		1 (Ref.)		1 (Ref.)	
Pos	242,886	15	1,946,100.51	0.007707721	2.155 (1.282, 3.621)		1.588 (0.937, 2.691)		2.141 (1.224, 3.746)		0.312 (0.043, 2.246)		1.670 (0.845, 3.297)		1.378 (0.597, 3.176)	
Migraine						0.163		0.3214		0.3133		0.6414		0.2153		0.9591
No	9,388,515	282	77,287,594.64	0.00364871	1 (Ref.)		1 (Ref.)		1 (Ref.)		1 (Ref.)		1 (Ref.)		1 (Ref.)	
Yes	265,939	12	2,189,141.9	0.0054816	1.509 (0.847, 2.688)		1.343 (0.750, 2.405)		1.523 (0.672, 3.452)		1.217 (0.533, 2.781)		1.572 (0.769, 3.215)		1.027 (0.376, 2.801)	
Depression						0.7611		0.2681		0.6598		0.3001		0.5353		0.3629
No	9,347,024	284	76,995,043.88	0.003688549	1 (Ref.)		1 (Ref.)		1 (Ref.)		1 (Ref.)		1 (Ref.)		1 (Ref.)	
Yes	307,430	10	2,481,692.66	0.004029508	1.103 (0.587, 2.072)		0.698 (0.369, 1.319)		0.832 (0.366, 1.890)		0.587 (0.215, 1.607)		0.753 (0.307, 1.847)		0.658 (0.267, 1.622)	
Psoriasis						<0.0001		0.0004		0.0027		0.1022		0.141		0.0007
No	9,592,144	286	78,967,421.5	0.003622	1 (Ref.)		1 (Ref.)		1 (Ref.)		1 (Ref.)		1 (Ref.)		1 (Ref.)	
Yes	62,310	8	509,315.04	0.015707	4.346 (2.153, 8.774)		3.572 (1.768, 7.219)		3.481 (1.541, 7.861)		3.207 (0.793, 12.976)		2.357 (0.753, 7.382)		4.734 (1.925, 11.641)	

Abbreviations: BMI—body mass index; CKD—chronic kidney disease; DM—diabetes mellitus; HR—hazard ratio; HTN—hypertension; IFG—impaired fasting glucose; IR—incidence rate (per 1000); TC—total cholesterol.

**Table 3:** HRs and 95% CI for the association between Kaposi sarcoma and common skin disorders.

		N	Event	Duration, PY	IR, 1000 PY	HR (95% C.I)			
						Model 1	Model 2	Model 3	Model 4
Atopic dermatitis	No	9,585,897	291	78,916,418	0.003687446	1 (ref.)	1 (ref.)	1 (ref.)	1 (ref.)
	Yes	68,557	3	560,318	0.0053541	1.459 (0.469, 4.542)	1.274 (0.408, 3.973)	1.260 (0.404, 3.932)	1.248 (0.400, 3.896)
	*p*-value					0.5145	0.6769	0.6904	0.7023
Dry skin	No	9,582,262	288	78,893,259	0.003651	1 (ref.)	1 (ref.)	1 (ref.)	1 (ref.)
	Yes	72,192	6	583,477	0.010283	2.835 (1.263, 6.363)	1.863 (0.828, 4.191)	1.850 (0.822, 4.161)	1.846 (0.821, 4.152)
	*p*-value					0.0115	0.1324	0.137	0.1384
Urticaria	No	9,314,372	275	76,693,150	0.003585718	1 (ref.)	1 (ref.)	1 (ref.)	1 (ref.)
	Yes	340,082	19	2,783,587	0.006825726	1.913 (1.202, 3.045)	1.475 (0.925, 2.353)	1.463 (0.917, 2.334)	1.460 (0.915, 2.329)
	*p*-value					0.0063	0.1029	0.1103	0.1125
Psoriasis	No	9,592,144	286	78,967,422	0.003622	1 (ref.)	1 (ref.)	1 (ref.)	1 (ref.)
	Yes	62,310	8	509,315	0.015707	4.346 (2.153, 8.774)	3.629 (1.797, 7.331)	3.603 (1.783, 7.280)	3.569 (1.766, 7.213)
	*p*-value					<0.0001	0.0003	0.0004	0.0004
Overall Comorbidity	No	9,155,245	263	75,395,398	0.003488	1 (ref.)	1 (ref.)	1 (ref.)	1 (ref.)
	Yes	499,209	31	4,081,338	0.007596	2.188 (1.508, 3.174)	1.699 (1.168, 2.470)	1.685 (1.159, 2.450)	1.679 (1.154, 2.441)
	*p*-value					<0.0001	0.0055	0.0063	0.0067

Overall comorbidity is that any of the four common skin disorders were present. Multiple regression analysis controlled by Model 1 (non-adjusted), Model 2 (age, sex), Model 3 (age, sex, BMI, income, smoking, drinking, regular exercise), and Model 4 (age, sex, BMI, income, smoking, drinking, regular exercise, DM, HTN, dyslipidemia).

## Data Availability

Access to individual participant data will not be granted, as it necessitates legitimate administrative approval. Data access is restricted in accordance with government regulations.
